# Pediatric Distal Tibia Physeal Injury Presenting with Acute In-toeing: A Case Report and Literature Review

**DOI:** 10.5435/JAAOSGlobal-D-22-00134

**Published:** 2023-08-02

**Authors:** Clark Yin, E. Graham Englert, Ricky Sareini, Ehab Saleh

**Affiliations:** From the Department of Orthopaedic Surgery, William Beaumont Hospital, Royal Oak, MI (Dr. Yin, Dr. Englert, and Dr. Saleh) and Wayne State University, School of Medicine, Detroit, MI (Dr. Sareini).

## Abstract

Ankle fractures are among the most common fractures sustained in the pediatric population. Given the frequency of physeal involvement of the distal fragment, complications including growth arrest, overgrowth, and rotational deformities are not uncommon. This case report describes a 12-year-old adolescent boy who presented after an acute right ankle injury sustained while playing. He noted right ankle pain, swelling, and in-toeing of his foot. Radiographs of the ankle demonstrated a distal tibia Salter-Harris type II fracture that appeared nondisplaced. However, a CT scan of the ankle demonstrated a 60° difference in the rotational profile between the injured and noninjured tibias. The patient's acute rotational deformity was corrected with closed reduction and percutaneous pinning. Pediatric distal tibia physis fractures presenting with in-toeing are rare and difficult to diagnose accurately with radiographs alone. Accordingly, a detailed history, physical examination, comparison radiographs, and CT scans are imperative in making the correct diagnosis and determining the appropriate treatment.

Ankle fractures are among the most common fractures sustained in the pediatric population.^1^ The distal tibia is the second most common site of fracture involving an epiphyseal plate. This injury has a particularly high complication rate including physeal bar formation, angular deformity, and articular incongruity.^2-4^ The physiologic epiphysiodesis that occurs over an 18-month period during adolescence predisposes these individuals to age-specific fracture patterns. The distal tibia ossification center closes starting centrally, then medially, and finally laterally. This process typically concludes at 15 years of age for female patients and 17 for male; however, there is much variation depending on the skeletal age.^5^

In 1968, Salter and Harris^6^ published their widely used physeal fracture classification system based on fracture pattern and prognosis. Two common transitional fractures that occur in adolescents are triplane and juvenile Tillaux fractures. Triplane fractures are SH IV fractures that are named after their involvement of three separate planes. Lateral triplane fractures occur from supination-external rotation injuries while medial triplane fractures result from adduction injuries.^5,7^ Tillaux fractures occur near skeletal maturity when only the anterolateral distal tibia physis remains unfused. A supination-external rotation injury can lead to an SH III avulsion of the anterior inferior tibiofibular ligament from the distal tibia.^5,7^ Although triplane and juvenile Tillaux fractures are common in adolescent patients, not all ankle fractures that occur in this age group can be classified as one of these two.

Adolescents with Tillaux, triplane, and other distal tibia physeal fractures commonly undergo closed reduction in the emergency department followed by a CT scan. Advanced imaging can be valuable in identifying the fracture morphology and determining the amount of articular step-off. Nonsurgical management in a cast is typically preferred in patients with less than 2 mm articular step-off. Surgical management consists of closed reduction and percutaneous pinning versus open reduction and internal fixation using screws depending on fracture morphology and proximity to skeletal maturity.^7^

Despite the high incidence of ankle fractures in pediatric patients, scarce literature describes the rotational deformity that can occur with these injuries. Pediatric distal tibia physeal injury presenting with an acute internal rotational deformity has not been reported before. In this report, the case of a 12-year-old adolescent boy who presented with acute in-toeing because of a distal tibia physeal injury is described, with a review of the literature.

## Case Report

**Figure 1:**
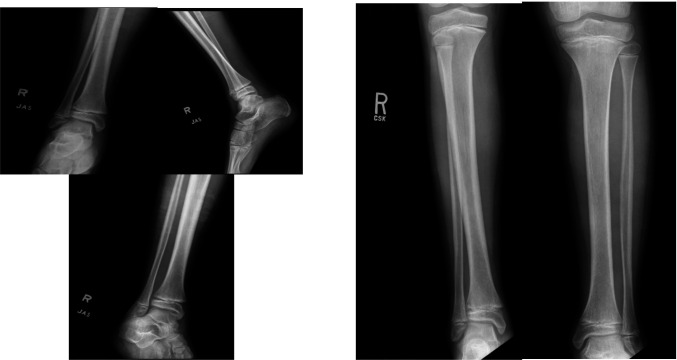
Initial radiographs of the patient's distal tibia. A Salter-Harris II fracture can be seen on the lateral radiograph. No obvious rotational deformity is appreciable on these images. The ankle mortise appears intact. The AP full-length radiographs of the right and left tibia demonstrate asymmetry.

A healthy 12-year-old adolescent boy presented to the office with right ankle pain and in-toeing after being dropped by his friend from standing height. The patient and his family denied prior rotational deformity or pain. On physical examination, there was mild right ankle swelling and tenderness to the medial ankle. His right thigh foot angle was measured at 35° of internal rotation compared with 25° of external rotation to the contralateral extremity. Radiographs of the right ankle and tibia and fibula revealed a distal tibia Salter-Harris type II fracture with physeal widening (Figure 1). Comparison views demonstrated an abnormal overlap between the tibia and fibula in the affected extremity, suggesting the presence of rotational displacement.

**Figure 2:**
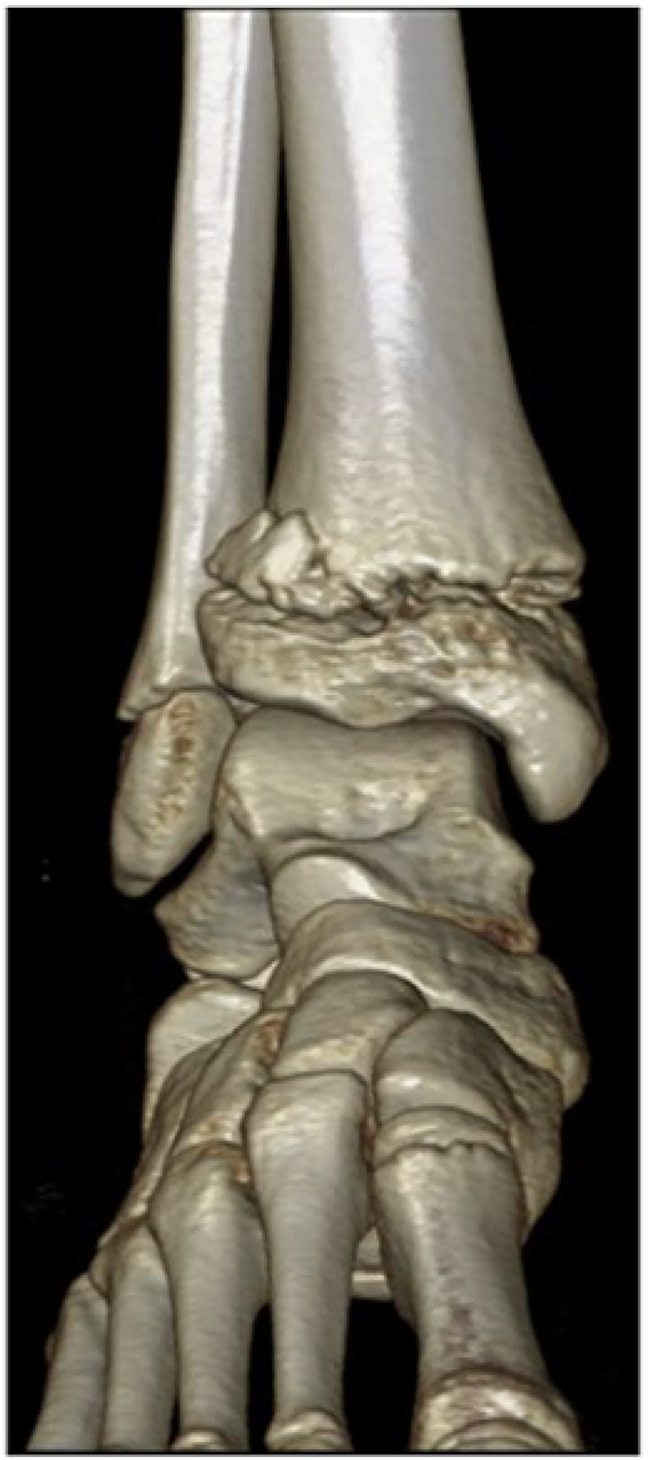
3D reconstruction image of the patient's distal tibia with a Salter-Harris II fracture.

An ankle CT scan with 3D reconstruction was obtained on the same day, which confirmed mismatch between the distal tibia metaphysis and epiphysis, confirming the rotational nature of the injury (Figure 2).

**Figure 3:**
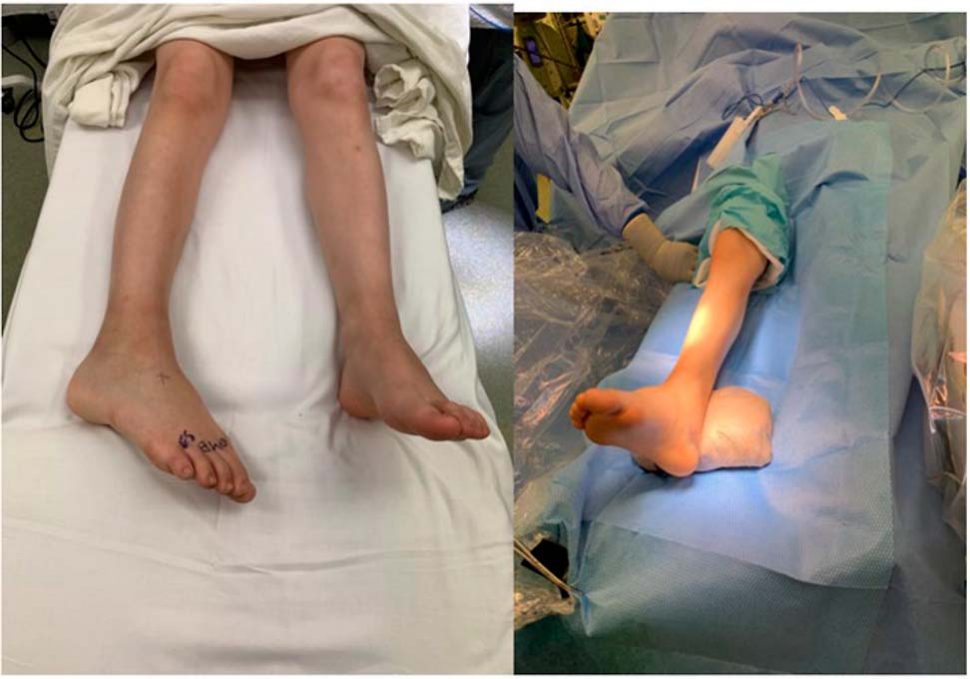
Preoperative and intraoperative images. With both knees pointing forward, the patient's right ankle is internally rotated 35°. Immediately after the reduction maneuver, the patient's deformity has been corrected.

**Figure 4:**
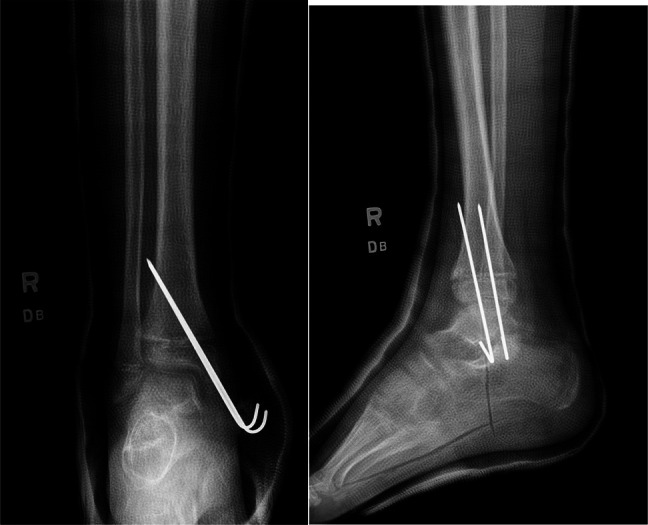
Radiographs showing AP and lateral views of percutaneous pinning.

The patient was scheduled for closed reduction and pinning versus open reduction and pinning. Under general anesthesia, the patient was positioned supine and the overlying skin was prepped and draped in a sterile fashion. Fracture reduction was obtained under image intensifier control with longitudinal traction and external rotation of the foot while maintaining the position of the tibia. After reduction, clinically the rotation of the right foot matched that of the contralateral side (Figure 3). The fracture was then pinned with smooth Kirschner wires inserted from the medial aspect of the distal tibia through the physis into the lateral cortex into the metaphysis. Radiographs confirmed good reduction and pin placement (Figure 4). The wires were bent and cut outside the skin; a dressing was applied; and the patient was fitted with a short leg univalved cast. The patient was discharged home on the same day.

## Follow-Up

**Figure 5:**
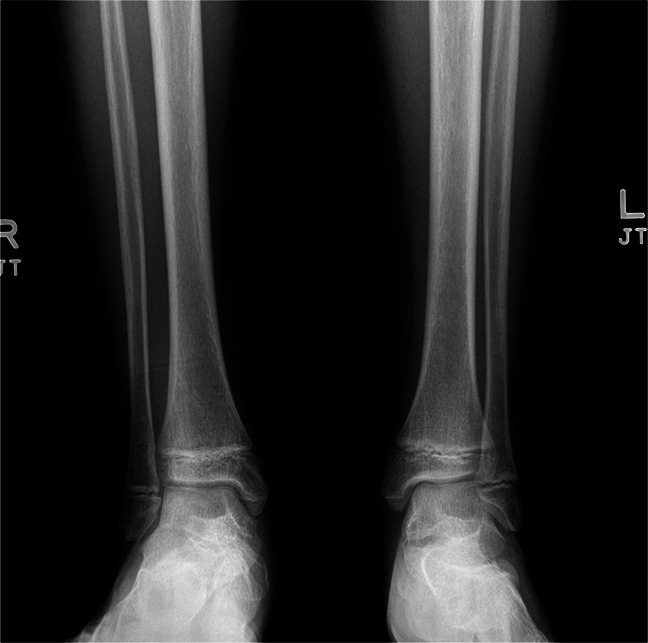
Radiograph showing an AP standing view of bilateral ankles at the 6-month postoperative visit demonstrating an open distal tibia physis on the right.

At 4 weeks postoperatively, the pins were removed and the patient continued to be non–weight-bearing in a cast. At 6 weeks, the cast was removed and the patient was allowed full weight-bearing and was instructed on ankle range of motion, strengthening, and stretching exercise programs. The patient resumed full activity at 3 months postoperatively. At the 6-month follow-up visit, he resumed all activities, denied ankle pain, and had full range of motion. His distal tibia physis remains open with no visible growth arrest or physeal bars (Figure 5).

## Discussion

Literature describing acute rotational deformities after distal tibial physeal fractures is rare and limited to patients presenting with external rotational deformities after acute distal tibia physeal injuries.^14,15^ This injury pattern is due to the common external rotation mechanism of injury. Nenopoulos et al^8^ described nine patients who sustained abrupt external rotation ankle injuries and subsequent external rotation deformity ranging from 20 to 40°. Most of their patients also sustained spiral fractures of the fibula. Of note, four patients in this series were misdiagnosed as a ‘strain’ or fracture of the fibula after their initial presentation. Four of their patients later experienced early closure of the distal tibia physis, but no leg length discrepancy was noted. Persistent external rotation deformity of 10 to 15° was noted in three patients at final follow-up. The authors concluded that rotational deformities can easily be misdiagnosed and that clinicians must be vigilant in looking for the radiological finding of an ‘open fish mouth’ on lateral view radiographs.^9^

Broock and Greer described a 7-year-old girl who sustained an external rotation injury, in which the foot was rotated 45° in relation to both malleoli. Radiographs demonstrated irregularity of the epiphyseal plate with no metaphyseal fracture. She was treated with closed reduction with traction and internal reduction with subsequent immobilization. When reproducing this injury with an anatomical dissection, the authors discovered that the distal tibial epiphysis was unable to be rotated until the entire circumference of the periosteum and perichondrium was elevated from their attachments.^10^

Perhaps the earliest published case of a purely rotational deformity was by Lovell^11^ in 1968 describing a 13-year-old adolescent boy who was injured while tobogganing. He proposed the injury was a posterior dislocation of the fibula with external rotation of the foot, but this was likely a rotational fracture of the distal tibial plate. Likewise, Nevelos et al reported an 11-year-old boy who sustained an external rotation injury, similarly treated with closed reduction and immobilization. At 12-month follow-up, no evidence of abnormality was noted when compared with the contralateral side.^12^

Although past literature describing acute rotational deformity has been limited to external rotation, there is one article that described internal rotation deformities as a sequela of pediatric distal tibia fractures. Moon et al^13^ described nine patients who sustained supination-inversion injury with an intact fibula. The average age at injury was 7.5 years, and the progressive deformity was observed over an average of 10 years after the injury. There were 2 cases of Salter-Harris type I fracture, 2 cases of type II, 1 case of type III, and 4 cases of type IV. Each of the patients underwent open reduction and Kirschner wire fixation. While these patients had concomitant varus deformities, they had an average of 23° of internal rotation deformity at final follow-up. Each of these patients had early closure of the medial distal tibial physis while the fibula continued to grow, resulting in disabling deformities to these ankles. The authors suggested that some intervention, even repeated corrective osteotomies, should be done to avoid this devastating outcome.

Rotational injuries are rare and can be easily missed on plain radiographs. Accordingly, it is imperative to clinically assess the rotation of the patient's injured extremity. Although our patient's in-toeing was impressive, more subtle rotational deformities may be difficult to appreciate. For this reason, we recommend a detailed history of rotational deformities and examining the rotational profile of the contralateral extremity. Providers must also have a low threshold to obtain contralateral extremity radiographs and advanced imaging of the injured ankle to better characterize any rotational deformity. We recommend percutaneous pinning or internal fixation after reduced distal tibial physeal rotational injuries. As with all injuries of epiphyseal plates, we plan to follow this patient until maturity.

## Conclusion

Isolated, acute internal rotation deformity after pediatric ankle fractures is a rare occurrence that has yet to be described. Previous cases of isolated rotational deformities about the distal tibia in the literature involved external rotation. These injuries can easily be overlooked, and undertreated; thus, a detailed history of prior deformity and examination of the contralateral extremity are critical for diagnosis. Successful treatment involves closed reduction and percutaneous pinning and/or internal fixation to avoid devastating ankle deformities. It is important to counsel patients and their families regarding the possibility of premature physeal closure and the necessity of close follow-up.

